# *TMC6/8*-associated epidermodysplasia verruciformis: germline variants and a complex structural alteration in a skin cancer predisposition syndrome

**DOI:** 10.1038/s41431-026-02043-8

**Published:** 2026-02-18

**Authors:** Ceren Damla Durmaz, Naz Güleray Lafcı, Dilsu Dicle Erkan, Ömer Çağrı Akçin, Nesibe Bulut, Fatih Kuş, Deniz Ateş Özdemir, Jürgen Neesen, Paul Dremsek, Ömer Dizdar

**Affiliations:** 1 Department of Medical Genetics, Faculty of Medicine, Hacettepe University, Ankara, Turkey; 2 Institute of Medical Genetics, Centre for Pathobiochemistry and Genetics, Medical University of Vienna, Vienna, Austria; 3 Department of Medical Oncology, Hacettepe University Cancer Institute, Ankara, Turkey; 4 Department of Medical Pathology, Faculty of Medicine, Hacettepe University, Ankara, Turkey

**Keywords:** Cancer genetics, Cancer genetics

## Abstract

Hereditary epidermodysplasia verruciformis (EV) represents a paradigmatic inherited cutaneous syndrome linking viral susceptibility, immunity, and oncogenesis. Although biallelic variants in *CIB1*, *TMC6*, and *TMC8*—encoding components of the keratinocyte-intrinsic antiviral complex—underlie most cases, the full mutational spectrum and its oncologic implications remain incompletely defined. We performed integrated genomic, histopathological, and longitudinal clinical analyses in six affected individuals from five unrelated families with confirmed hereditary EV. Comprehensive short-read sequencing, copy-number assessment, and optical genome mapping (OGM) were used to delineate the underlying genetic alterations, followed by long-range PCR and Sanger validation. Pathogenic or likely pathogenic germline variants affecting *TMC6* or *TMC8* were identified in all probands, providing molecular confirmation of disease. Four variants were novel, including splice-site, frameshift, and in-frame deletions. In one proband, OGM revealed a previously unrecognised complex del–inv–del structural variant spanning both *TMC6* and *TMC8*—the first reported example in hereditary EV. This rearrangement, confirmed at base-pair resolution, co-segregated with a synonymous *TMC8* variant that served as a practical haplotypic marker for carrier testing. Clinically, all patients developed cutaneous squamous cell carcinoma (SCC), and several exhibited multifocal or aggressive disease, underscoring the deterministic malignant potential of hereditary EV. This study broadens the genetic and phenotypic spectrum of *TMC6/TMC8*-associated EV, establishes complex structural rearrangement in the molecular etiology, and consolidates hereditary EV as a recessive cancer predisposition syndrome. Integrating high-resolution genome mapping into diagnostic workflows may uncover concealed allelic architecture in unresolved hereditary cancer syndromes.

## Introduction

Hereditary cancer syndromes account for approximately 5–10% of all malignancies [1]. They are among the most clinically actionable entities in oncology because their identifiable germline basis enables targeted surveillance and prevention strategies. While dominantly inherited syndromes such as Li-Fraumeni syndrome [2], Lynch syndrome [3], and familial adenomatous polyposis [4] are well characterised and have clear clinical guidelines, recessive cancer predisposition syndromes are less well recognised. Among them are syndromic DNA repair disorders such as xeroderma pigmentosum [5], Fanconi anaemia, Bloom syndrome, and ataxia–telangiectasia [6], which are usually identified because of their systemic features, including neurodevelopmental delay, immunodeficiency, or bone marrow failure. There are also non-syndromic recessive disorders, including *MUTYH*- and *NTHL1*-associated polyposis [7, 8], that present solely with tumour predisposition. These non-syndromic forms are very rare and may be underdiagnosed due to the absence of syndromic stigmata.

Among these, epidermodysplasia verruciformis (EV; OMIM 226400, 618231, 618267) is an exceptionally rare genodermatosis and a paradigmatic example of recessive cancer susceptibility. Clinically, EV is characterised by early-onset flat warts and pityriasis versicolor–like lesions, typically on sun-exposed sites, and predisposes to non-melanoma skin cancers, most prominently cutaneous squamous cell carcinoma (SCC), which develops in 30–70% of affected individuals [9–12]. EV is now recognised as a heterogeneous entity with three distinct forms: (i) typical/hereditary EV, caused by biallelic pathogenic variants in *TMC6* (EVER1), *TMC8* (EVER2), or *CIB1* [13, 14]; (ii) atypical EV, arising in the context of broader immunodeficiency syndromes such as *DOCK8* deficiency or WHIM syndrome [15]; and (iii) acquired EV, observed under secondary immunosuppression including HIV infection or post-transplant therapy [16]. Among these, the canonical hereditary form (typical EV) is by far the rarest. According to HGMD Professional (version 2024.4), 24 pathogenic/likely pathogenic variants have been reported in *TMC6*, 22 in *TMC8*, and 11 in *CIB1* to date [17].

From a mechanistic perspective, hereditary EV results from the disruption of a keratinocyte-intrinsic antiviral complex composed of TMC6, TMC8, and CIB1 located on the endoplasmic reticulum membrane and nuclear envelope [14]. The complex acts as a restriction factor against β-human papillomavirus (β-HPV). Loss of either TMC6 or TMC8 destabilises CIB1, which is normally protected from ubiquitination and degradation through its interaction with the TMC proteins [18]. Breakdown of the complex permits persistent β-HPV infection and lesion formation. Additionally, TMC6 and TMC8 interact with the zinc transporter ZnT-1 independently of CIB1, thereby maintaining intracellular zinc balance [19]. Disruption of this pathway increases nuclear zinc, activates AP-1 and NF-κB, and creates a permissive environment for β-HPV replication [20]. Chronic viral infection, together with ultraviolet-induced DNA damage, drives the development of cutaneous SCC, characteristic of EV [21]. However, recent functional studies have begun to challenge this strictly keratinocyte-intrinsic model. Experimental work in mouse models and tissue culture systems suggests that the antiviral and tumor-suppressive functions of the TMC6–TMC8–CIB1 complex may not be confined to keratinocytes but may instead depend on non-keratinocyte activities, potentially involving broader immune-mediated mechanisms. While the precise contribution of epithelial versus systemic immune functions remains to be fully resolved, these findings highlight an evolving understanding of EV susceptibility that extends beyond a purely cell-autonomous defect [22–24].

Here, we studied six affected individuals from five unrelated Turkish families with genetically confirmed typical EV. Our series is distinguished by its detailed clinical documentation, high oncological burden with multiple recurrent SCCs, and its expansion of the mutational spectrum through the identification of four novel variants. Importantly, we describe the first complex *TMC6/TMC8* rearrangement resolved by OGM, highlighting structural variation as an under-recognised pathogenic mechanism in EV. Together, these findings provide novel insights into the clinical and molecular spectrum of hereditary EV and emphasise the need for comprehensive genomic approaches to secure accurate diagnosis and inform patient management.

## Materials and methods

### Study group

This study involved six affected Turkish individuals from five unrelated families diagnosed with the hereditary form of epidermodysplasia verruciformis. All affected individuals were evaluated within a multidisciplinary setting, including the Departments of Medical Genetics, Medical Oncology, and Medical Pathology. Clinical assessments were carried out by clinical geneticists and oncologists. Demographic details, age at onset of lesions, lesion distribution, family history including consanguinity and affected relatives, as well as prior medical and surgical treatments, were retrieved from medical records. Oncological information encompassed the occurrence and anatomical localisation of NMSCs, recurrence rates, the number of excisions performed, and overall clinical course. Available skin biopsies were reviewed by a dermatopathologist, and histopathological findings were integrated with clinical and genetic data.

### Genetic analysis

Genomic DNA was isolated from peripheral blood samples of the probands and affected family members using the Wizard™ Genomic DNA Purification Kit (Promega, Madison, WI, USA) according to the manufacturer’s instructions. Initial testing consisted of Sanger sequencing of the canonical EV genes. In an unresolved case (P6), broader genomic approaches such as clinical exome sequencing (CES) and OGM were subsequently applied.

#### Sanger sequencing

All coding exons and exon–intron boundaries of *TMC6*, *TMC8*, and *CIB1* were PCR-amplified and subsequently sequenced by Sanger sequencing on an ABI 3500 Genetic Analyser (Thermo Fisher Scientific, Waltham, MA, USA). Primer sequences are available upon request. In proband P6, where OGM had identified a complex structural variant involving both *TMC6* and *TMC8* genes (17q25), breakpoint-spanning long-range PCR was carried out using Takara LA Taq® polymerase with primers designed on OGM-defined junctions to orthogonally validate the complex rearrangement.

#### Next-generation sequencing

For proband P6, Sanger sequencing identified only a single heterozygous variant, although the clinical presentation was highly suggestive of epidermodysplasia verruciformis. CES was therefore undertaken to search for a second pathogenic allele not detected by targeted testing. Library preparation was carried out using the KAPA HyperCap Heredity Panel (Roche, Basel, Switzerland), which targets approximately 10 Mb across 3332 genes associated with hereditary disorders, including those implicated in monogenic forms of persistent human papillomavirus infection [25]. Sequencing was undertaken on a DNBSEQ-G400 platform (MGI, Shenzhen, China). Bioinformatic analysis was performed with the SEQ Platform (Genomize, v.9.0, Istanbul, Turkey), a secure web-based clinical genomics software environment providing an end-to-end analysis pipeline. Copy-number variant (CNV) analysis was also integrated in the SEQ platform (Genomize, v9.0), combining the GATK CNV algorithm [26] with additional split-read support from Delly [27]. CNVs recurrently observed across unrelated samples in the same sequencing run were excluded as likely artefacts.

#### OGM

OGM was performed to further characterise the potential structural variant (SV) in proband P6. Ultra–high–molecular–weight (UHMW) DNA was extracted from frozen peripheral blood using the Bionano Prep SP-G2 Frozen Human Blood DNA Isolation Kit (Bionano Genomics, San Diego, CA, USA) according to the manufacturer’s instructions. Briefly, leukocytes were quantified using a HemoCue WBC Analyser (HemoCue, Ängelholm, Sweden), and approximately 1.5 × 10⁶ viable cells were processed for DNA isolation. Following lysis with detergents and proteinase K, DNA was precipitated with isopropanol and captured on a magnetic disk, then washed, eluted, and equilibrated overnight at room temperature.

DNA labelling was performed using the Bionano Direct Label and Stain (DLS-G2) Kit (Bionano Genomics, San Diego, CA, USA) as previously described, and labelled molecules were loaded onto a Saphyr G3.3 Chip for nanochannel linearization and high-resolution imaging on the Saphyr System (Bionano Genomics, San Diego, CA, USA) [28]. Data collection was targeted at approximately 1300 Gbp of UHMW DNA (including molecules ≥ 150 kb).

De novo analysis (aligned to GRCh38) and variant annotation were performed with the Bionano Access Suite (v1.8.2) with the respective software modules. SV and CNV calling were conducted using the manufacturer’s default algorithms—one for breakpoint-based SV detection and another for CNV identification based on molecule coverage—without frequency-based filtering. Automated calls were cross-checked through manual curation focused on the 17q25 locus, with direct visual inspection of the genomic region of interest by an experienced analyst to ensure accurate breakpoint resolution and exclude potential false positives.

#### Variant interpretation

All single-nucleotide variants, small insertions/deletions, and splice-site alterations identified by Sanger sequencing or CES were evaluated according to the American College of Medical Genetics and Genomics (ACMG) 2015 guidelines [29]. CNVs and SVs detected by OGM were assessed based on the ACMG/ClinGen 2020 technical standards for CNV interpretation and integrated case-specific criteria [30]. Variant annotation included cross-referencing with population and clinical databases such as gnomAD [31], ClinVar [32], and VarSome [33]. CNVs were additionally curated using ClinGen Dosage Sensitivity Map [30] and DECIPHER [34]. Splice-site variants were analysed using Human Splicing Finder (HSF) [35], in-frame deletions with the Ensembl Variant Effect Predictor [36], and genomic breakpoints overlapping repetitive elements were screened using RepeatMasker [37] to identify potential sequence motifs associated with structural instability.

### Writing assistance

The authors used an AI-based writing assistant (ChatGPT, OpenAI) for language editing support. All sections of the manuscript were subsequently checked and approved by the authors.

## Results

### Cohort overview and clinical features

Six affected individuals (three females, three males) from five unrelated Turkish families with hereditary EV (Table 1) were studied. Parental consanguinity was present in four of the five families, while proband P6 was the only individual born to non-consanguineous parents. Family 2 included two affected sisters (P2, P3) whose deceased brother had also exhibited a similar clinical phenotype consistent with EV.

**Table 1:** Clinical, pathological, and genetic findings of the affected individuals in this study.

Patient ID	P1	P2	P3	P4	P5	P6
**Family**	F1	F2		F3	F4	F5
**Age**	44	36	34	63	74	50
**Sex**	F	F	F	M	M	M
**Consanguinity**	+	+	+	+	+	–
**Family history of EV**	–	+	+	–	–	–
**Age of onset for EV**	25	15	15	22	6	39
**Localisation of lesions**	H, N, E	H, N, L, E	H, N, T, E, G	H, N, E	H, E	H, N, E
**SCC/BCC**	+/–	+/–	+/–	+/+	+/+	+/+
**SCC/BCC Stage**	1/-	2/–	3/–	3/1	2/–	3/1
**SCC/BCC diagnosis count**	3/-	1/–	multiple/-	4/1	4/1	1/1
**Enucleation**	+	+	–	–	–	–
Chemo/Radiotherapy*	–/+	+/–	–/–	+/+	+/–	–/–
Genetic Variant**	*TMC6*:c.559_567del p.(Gly187_Tyr189del) ***	*TMC8*:c.1127+1 G > C ***		*TMC6*:c.1716-1 G > A***	*TMC6*:c.686del p.(Pro229ArgfsTer6)***	*TMC8*:c.1127+1 G > C♦
						*del-inv-del*♦
**Tumour Grade/Stage**	2/pT1	3/pT3	2/pT4	2/pT1	2/pT4	2/pT3
**Lymphovascular Invasion**	–	–	+	–	–	+
**Perineural Invasion**	–	–	+	–	–	–
**Breslow Thickness**	3	14	7.5	20	21	4.5
**Clark Level**	III	V	IV	III	V	V
**Remission**	+	–	–	+	–	+
**Follow-up (months)**	34	129	142	53	149	101
**Vital status**	Alive	Alive	Alive	Alive	Deceased (at age 74)	Alive
**Last Follow-up (date)**	September, 2025	April, 2025	May, 2025	April, 2024	–	February, 2025

*F* Female, *M* Male, *H* head, *N* neck, *L* laryngeal, *T* truncal, *E* extremities, *G* genital.

*RT was administered before the molecular diagnosis.

**written according to *TMC8* NM_152468.5, *TMC6* NM_001127198.5 transcripts.

Homozygous mutations are indicated by ***, and the compound heterozygous mutation by ♦.

**Fig. 1:**
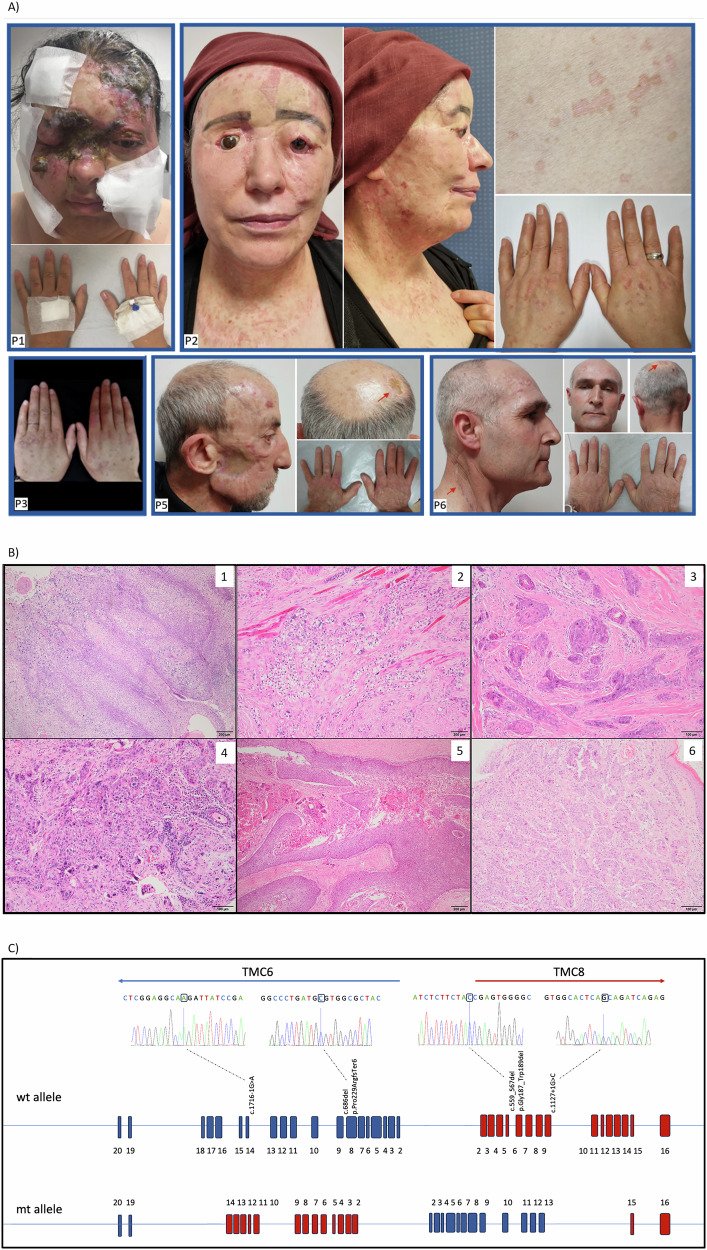
Clinical, histopathological, and molecular spectrum of hereditary epidermodysplasia verruciformis (EV) in six affected individuals. **A** EV and SCC/BCC lesions on sun-exposed areas of the affected individuals. **B** Histopathological features of the squamous cell carcinomas in the affected individuals. 1. Perinuclear clearing resembling koilocytosis in malignant squamous epithelial cells in the tumor from P1’s cheek (H&E, 100x). 2. Clear cell neoplasia infiltrating between striated muscles in the biopsy from P2’s cheek (H&E, 100x). 3 and 4: Infiltrative islands with large nuclei and abundant eosinophilic cytoplasm in malignant squamous cells from P3’s sternum and P4’s lip (H&E, 100x-C, 40x-D) 5: Infiltrative islands containing cystic spaces in P5’s scalp biopsy (H&E, 100x) 6: Infiltrating squamous cell islands in the dermis of P6. **C** Schematic representation of the *TMC6* and *TMC8* loci and variants identified in this study. Coding exons of *TMC6* and *TMC8*, located on chromosome 17q25, are shown schematically. *TMC6* is transcribed from the reverse (minus) strand, whereas *TMC8* is oriented on the positive strand. Single-nucleotide variants identified in this study are mapped onto the wild-type (wt) allele, with the corresponding Sanger electropherograms displayed above each variant. The complex *TMC6/TMC8* structural rearrangement identified in proband P6 is illustrated in the lower panel (mutant allele).

Cutaneous manifestations were characterised by early-onset flat warts and pityriasis versicolor–like lesions, with a median age of onset of 20 years (range 6–39). Lesions predominantly involved sun-exposed regions (head and neck), and in several patients extended to the trunk, extremities, genital region and even laryngeal mucosa, indicating generalised involvement (Fig. 1A). There was no known underlying congenital or acquired immunodeficiency that could explain the aggressive HPV infections observed in these individuals.

A substantial tumour burden was observed across the cohort. All six patients developed cutaneous SCC, frequently multifocal and/or recurrent. Basal cell carcinoma (BCC) occurred in three patients (P4–P6). Eye enucleation was required in two patients (P1, P2) due to ocular involvement. Histopathological review confirmed high-grade tumours with Clark levels of SCC ranging from III to V and Breslow thickness up to 21 mm in P5. Lymphovascular invasion was evident in P3 and P6, while perineural invasion was noted in P3. Representative histopathological features are shown in Fig. 1B, with detailed descriptions provided in the accompanying legend. In addition, proband P4 developed intrahepatic cholangiocarcinoma alongside metastatic SCC. Proband P6, one of the most severely affected individuals, presented with stage III cutaneous SCC (Clark level V), accompanied by metastatic spread to regional lymph nodes on PET-CT, necessitating adjuvant radiotherapy.

Therapeutic interventions included multiple surgical excisions across the cohort, with adjuvant chemotherapy or radiotherapy applied in selected cases. At the last follow-up, two patients were in remission, one had stable disease, and three had ongoing disease activity, reflecting the persistent and progressive clinical burden of hereditary EV. One proband (P5) succumbed to disease-related complications at the age of 74. The detailed clinical and molecular findings of the cohort are summarised in Table 1.

### Genetic findings

Molecular analysis revealed pathogenic or likely pathogenic variants in *TMC6* and *TMC8* in all six probands, thereby establishing a molecular diagnosis of typical EV in each case. Five distinct germline variants were identified across the cohort, including one frameshift, one in-frame deletion, two splice-site variants and one complex structural rearrangement involving *TMC6* and *TMC8* (Fig. 1C). Four of these variants were novel. Patients born to consanguineous parents harboured homozygous variants, whereas the non-consanguineous proband (P6) carried compound heterozygous alterations.

In proband P1, we identified a novel in-frame deletion (c.559_567del) on exon 6 of *TMC8* p.(Gly187_Tyr189del), which removes three amino acids from a predicted transmembrane domain. Structural modelling suggests that this subtle alteration may destabilise the domain and impair membrane anchoring of the EVER protein complex. In proband P5, a frameshift mutation in *TMC6* (c.686del; p.(Pro229ArgfsTer6)) on exon 8 was present in the homozygous state, introducing a premature termination codon and presumably resulting in complete loss of function through nonsense-mediated decay.

In two families, splice-site mutations were identified as the underlying cause. One patient (P4) carried a novel variant, *TMC6* c.1716-1G>A, affecting the intron 13–14 acceptor site and predicted by HSF to abolish the canonical splice acceptor signal. The recurrent variant on the intron 9-10 donor site of *TMC8* c.1127+1G>C, previously reported in the homozygous state in unrelated consanguineous families with early-onset EV [38], was also detected in two individuals (P2, P3) from one family (F2) in the homozygous state. The same splice mutation c.1127+1G>C was also identified in a heterozygous state in P6, together with a heterozygous intragenic structural variant affecting both *TMC6* and *TMC8*.

### Case vignette-P6

**Fig. 2:**
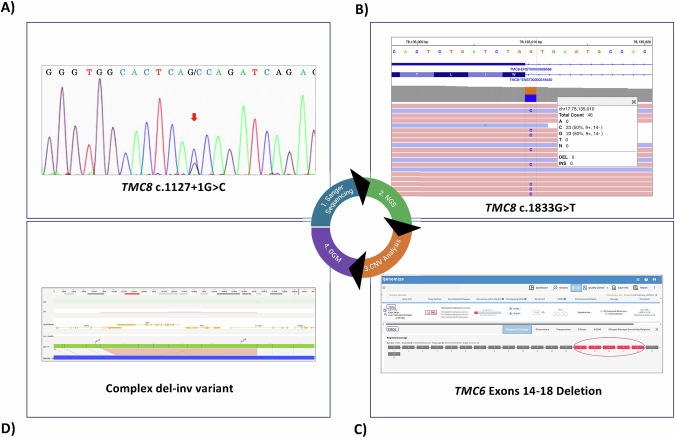
Sequential diagnostic workflow integrating complementary genomic approaches in proband P6. **A** Sanger sequencing showing heterozygous splice-site variant in *TMC8*. **B** Next-generation sequencing read alignment revealing a synonymous *TMC8* variant that was not detected by Sanger sequencing. **C** CNV analysis of CES identifying a heterozygous multi-exonic deletion in *TMC6*. **D** OGM resolving the second pathogenic allele as a complex deletion–inversion (del–inv) spanning *TMC6* and *TMC8*, which was further refined by breakpoint-spanning long-range PCR and Sanger sequencing as a del–inv–del configuration.

Proband P6 exemplified the diagnostic challenge posed by recessive disorders when routine sequencing identified only a single pathogenic allele (Fig. 2). Initial testing revealed a heterozygous splice-site variant in *TMC8* (c.1127+1G>C), the same mutation found in homozygous form in the two affected siblings from Family 2, but no second allele could be identified at this stage.

Given that only a single heterozygous variant had been identified by Sanger sequencing, CES was pursued to search for a second pathogenic allele. CES confirmed the heterozygous c.1127+1G>C variant and additionally identified a rare heterozygous synonymous change in *TMC8* (NM_152468.5: c.1833G>T, p.(Leu611=)), absent from population databases. Despite repeated attempts, this synonymous variant could not be consistently demonstrated by Sanger sequencing. The most plausible explanation is allele-specific amplification failure (allele drop-out), presumably resulting from an underlying structural alteration at this locus that prevents efficient amplification of the rearranged allele.

Concurrently, CNV analysis of CES data indicated a heterozygous deletion encompassing *TMC6* exons 14–18. Although this finding alone was insufficient to explain the phenotype, the genomic proximity of *TMC6* and *TMC8* raised the possibility of a larger rearrangement involving both genes.

**Fig. 3:**
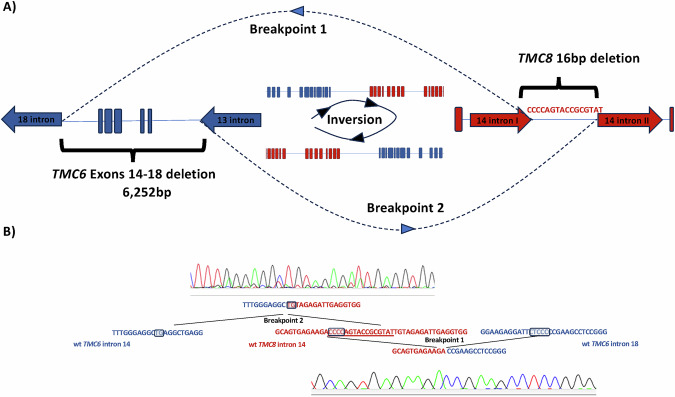
Overview of the complex del–inv–del rearrangement involving *TMC6* and *TMC8.* **A** Schematic representation of the hypothesized replication-based mechanism. Collapse of the replication fork and template switching at short homology tracts result in inversion, multi-exonic deletion of *TMC6*, and a 16 bp deletion within *TMC8*. **B** Partial electropherograms of the genomic region neighboring the rearrangement breakpoints, along with the breakpoint sequences of the complex del–inv–del variant. Wild-type *TMC8* intron 14, *TMC6* intron 14 and *p* intron 18 sequences are shown in the middle, with C-tracts and nucleotide TG highlighted. The breakpoint 1 junction demonstrates deletion of the intervening sequence (underlined) and fusion mediated by a CCCC/CCCCC microhomology motif. Microhomology between C-tracts facilitated deletion + junction formation. Sequence analysis of the breakpoint junctions revealed deletions in both junctions of 6,253 bp and 16 bp, respectively.

Based on these converging observations, OGM revealed that the rearrangement was not a simple deletion but a composite del–inv–del event, in which a large segment encompassing most of *TMC6* and *TMC8* was inverted, accompanied by a multi-exonic deletion of *TMC6* (exons 14–18) on one side and a 16 bp microdeletion at the *TMC8* breakpoint on the other. Breakpoint-spanning long-range PCR followed by Sanger sequencing confirmed the junctions at nucleotide resolution, thereby establishing the precise configuration of the complex rearrangement, which can be described according to the HGVS nomenclature as NC_000017.11:g.[78,114,160_78,120,412del;78,120,413_78,139,056inv;78,139,057_78,139,072del]. Even though RepeatMasker demonstrated an MLT1D element encompassing the breakpoint in intron 18 of *TMC6*, no repeat region was present in the counterpart breakpoint in *TMC8*. Notably, sequence similarities were detected at both breakpoints. Together, these findings support a composite mechanism involving both repeat-mediated recombination and replication-based template switching (Fig. 3). These results established compound heterozygosity for the pathogenic splice-site variant in *TMC8* (c.1127+1G>C) and a complex structural alteration involving *TMC6/TMC8*, thereby providing a definitive molecular diagnosis.

## Discussion

Epidermodysplasia verruciformis has long been recognised as a rare genodermatosis and a disease model at the intersection of viral infection, immunity, and carcinogenesis. While atypical and acquired EV forms observed in immunocompromised individuals demonstrate that the phenotype is not exclusively monogenic, hereditary EV remains the prototypical example of a recessively inherited disorder defined by keratinocyte-intrinsic immune dysfunction. Our cohort adds detailed clinical, histopathological, and molecular data from six affected individuals across five unrelated families.

Similar to other recessive cancer predisposition syndromes such as xeroderma pigmentosum, Bloom syndrome, or Fanconi anaemia, hereditary EV displays an almost inevitable malignant course; however, it is mechanistically distinct in being non–DNA repair-based and instead rooted in defective keratinocyte immunity [14]. The presence of β-HPV alone is likely insufficient to drive malignant transformation, as not all EV-associated cutaneous lesions, even those containing oncogenic HPV types, progress to cancer [11]. Instead, tumorigenesis in hereditary EV appears to arise from a multistep interplay between persistent viral infection and cumulative ultraviolet-induced damage. All affected individuals in our cohort developed cutaneous SCC, and three additionally developed BCC, underscoring the universal malignant potential of the disease. Lesions predominantly affected sun-exposed areas such as the head and neck, but in several patients extended to the trunk, extremities, genital skin, and notably the laryngeal mucosa—an unusual finding, as mucous membranes are known to be typically spared in EV [39]. The disease showed a broad clinical spectrum with variable expressivity: for example, in one sibling pair, both harbouring the same homozygous *TMC8* splice site mutation, the younger one developed multiple SCCs while the other manifested only a single lesion during follow-up.

Previous studies have suggested that individuals carrying *TMC6* variants tend to present with an earlier onset of lesions compared with those harbouring *TMC8* mutations [15]. Consistent with previous reports, proband 5 with a homozygous *TMC6* variant showed the earliest disease onset in our cohort, whereas this pattern was less evident in the second proband carrying a different *TMC6* variant. Conversely, late-onset cases have also been described, typically manifesting between the third and fourth decades of life [40, 41], and proband 6 in our series fits within this late-onset spectrum. This prolonged latency between infection onset and tumour development suggests a form of time-dependent penetrance, in which carcinogenesis reflects the gradual collapse of cutaneous immune surveillance rather than constitutive genomic instability [21].

Skin cancers in hereditary EV typically develop slowly but exhibit locally aggressive behaviour. In our cohort, disease severity was striking, with two patients requiring ocular enucleation because of locally invasive SCC, representing an exceptionally rare yet illustrative manifestation of the destructive potential of EV-associated malignancies.

Provided that cancers are treated appropriately, life expectancy in hereditary EV is generally comparable to that of the general population, although multifocal carcinomas may be fatal if left untreated [42]. In our cohort, one patient with multifocal SCC and concurrent BCC died from disease-related complications, whereas two probands with a single SCC showed divergent outcomes—one remained in remission while the other exhibited persistent disease activity.

The histopathological spectrum of EV-associated squamous carcinomas in our series paralleled the clinical aggressiveness of the disease. Tumours exhibited variable degrees of keratinisation, perinuclear clearing reminiscent of koilocytosis, and occasional clear-cell morphology, together with deeply infiltrative growth patterns. Such findings indicate that viral cytopathy and invasive transformation may coexist within the same lesion, supporting a model of multifocal, stepwise carcinogenesis in hereditary EV. Taken together with the high tumour burden and recurrence rate, these pathological findings substantiate that hereditary EV represents a cutaneous cancer predisposition syndrome rather than a benign viral dermatosis, highlighting the need for proactive surveillance.

Genetically, most reported cases of hereditary EV to date have been attributed to nonsense, frameshift, missense, or splice-site mutations, whereas SVs have rarely been systematically investigated [17]. In our cohort, we identified five distinct pathogenic variants, including splice-site, frameshift, and in-frame deletion mutations as well as a complex structural rearrangement, all of which, except for one splice-site variant, have not been previously reported in the literature.

The *TMC8* c.1127+1G>C splice-site variant identified in Family 2 (homozygous in two affected sisters) and present in the heterozygous state in proband P6 has also been reported in an unrelated Turkish family with hereditary EV without SCC [38]. Although formal relatedness between these families cannot be inferred from the available data, the recurrence of this rare allele across multiple families from the same national population raises the possibility of a shared ancestral origin. This variant has been shown to escape nonsense-mediated decay, producing aberrantly spliced yet stable transcripts [38]. This finding suggests that partial translation of such mutant transcripts may yield truncated TMC8 protein species that do not result in complete loss of function but rather disturb the stoichiometric balance of the CIB1–TMC6–TMC8 complex, thereby compromising the keratinocyte-intrinsic antiviral barrier. In our cohort, however, SCC occurred in all three individuals harboring this splice-site mutation, indicating that the lack of malignancy in previously reported cases may reflect shorter clinical follow-up or age-dependent penetrance rather than a truly attenuated oncogenic potential of this variant.

Proband 6 illustrated a distinct diagnostic path and initially posed a diagnostic challenge. As detailed in the results section, except for the heterozygous splice-site mutation in *TMC8*, no pathogenic variants in *CIB1*, *TMC6* or *TMC8* were detected by conventional Sanger sequencing despite a clinical phenotype highly suggestive of EV. Intriguingly, CES subsequently identified a rare synonymous variant (c.1833G>A) in *TMC8* in an apparently heterozygous state, accompanied by a heterozygous deletion encompassing *TMC6* exons 14–18 revealed by copy-number analysis of the short-read data. The persistent sequencing discrepancy, reflected in the repeated inability to validate the synonymous *TMC8* variant by Sanger sequencing with multiple primer pairs, raised suspicion of allelic dropout caused by an underlying complex structural variant. This observation prompted consideration of an alternative explanation that an undetected SV, invisible to Sanger sequencing and only partially indicated by CES, could be in cis with the synonymous change and represent the missing second pathogenic allele. This prompted further analyses using OGM and long-range PCR, which ultimately established the molecular diagnosis and reconciled the initial sequencing discrepancy.

OGM elucidated the SV hypothesized based on prior sequencing results, resolving it as a complex deletion–inversion spanning both *TMC6* and *TMC8* in cis on a single chromosome. Breakpoint-level sequencing revealed short microhomologies, consistent with a replication-based rearrangement mechanism such as fork stalling and template switching (FoSTeS) and microhomology-mediated break-induced replication [43, 44]. The proposed replication-based mechanism underlying this complex rearrangement is illustrated in Fig. 3 and discussed in greater detail in the Supplement.

Long-range PCR across the breakpoints inferred from the OGM data, followed by Sanger sequencing, confirmed both junctions and demonstrated that the synonymous *TMC8* variant resides in cis with the complex structural rearrangement. This finding explains its hemizygous readout in the initial Sanger sequencing and the apparent heterozygosity in CES. Collectively, these results indicate that the true second pathogenic allele was the complex del–inv–del structural variant rather than the synonymous substitution.

Once the del–inv–del rearrangement was resolved, the synonymous variant’s interpretation shifted from a candidate pathogenic allele to a haplotypic marker, functionally benign yet clinically valuable because it marks the structural allele in cis. This finding has practical consequences in families where direct SV genotyping through OGM or long-read sequencing is not feasible, as such a marker can serve as a proxy for reproductive counseling and carrier testing, analogous to established haplotype-based strategies used in other hereditary disorders [45]. The identified cryptic rearrangement disrupts both *TMC6* and *TMC8*. To date, no SV simultaneously affecting both genes has been reported. Only a single intragenic *TMC6* deletion has been documented [46]. Our findings, therefore, expand the mutational spectrum of EV and represent the first known instance of compound heterozygosity involving a splice-site mutation on one allele and a complex multi-gene SV on the other in this disorder. This observation emphasizes that cryptic pathogenic SVs may underlie clinically typical yet genetically unresolved forms of recessive hereditary disease [47–49], with EV serving as a prototypical example.

The identification of this complex SV also has broader implications for rare disease genomics [50–52]. However, conventional panel-based approaches such as CES and ES remain intrinsically limited in detecting such rearrangements, since most SV breakpoints lie within intronic or intergenic regions that are not captured by these assays. Nevertheless, as demonstrated in our case, careful interpretation of subtle analytical discrepancies in conventional short-read data can provide critical clues that prompt deeper structural investigation.

Nevertheless, this study has several limitations. The cohort size is necessarily small, as expected for an ultra-rare disorder. Functional analyses at the RNA or protein level were not performed, and tumor-based investigations such as HPV genotyping, viral transcript profiling, or somatic mutational analysis could not be included. Consequently, the interplay between germline predisposition, viral factors, and somatic events could not be explored in depth. Despite these limitations, by integrating clinical, histopathological, and genomic data, our study delineates the aggressive oncological course of hereditary epidermodysplasia verruciformis and makes several key contributions. It supports that hereditary EV constitutes a recessive cancer-predisposition syndrome characterised by a high tumour burden, multifocal invasive SCC, and marked histopathological diversity in which viral cytopathic changes coexist with infiltrative growth patterns. Although hereditary EV has distinctive molecular and pathological features, it remains substantially under-recognised in clinical practice. Early-onset, widespread HPV-related warts in immunocompetent individuals, particularly with family history or consanguinity, should prompt molecular testing. Heightened clinician awareness and early molecular testing are essential to ensure timely diagnosis, cancer surveillance, and appropriate genetic counselling in this rare yet clinically significant disorder.

Recognition of hereditary EV should prompt intensified dermatologic–oncologic surveillance. Although there is no curative therapy, disease progression can be controlled through photoprotection, regular skin checks, and timely biopsy followed by appropriate surgical intervention [53]. Equally important for oncologists unfamiliar with EV, radiotherapy should be avoided, as radiation exposure may accelerate tumour progression by enhancing local immunologic permissiveness within irradiated fields.

Lastly, our findings expand the mutational spectrum of hereditary EV to include complex SVs and demonstrate the diagnostic importance of incorporating the detection of such variants into genetic analysis. In particular, in autosomal-recessive disorders, if only one pathogenic variant is identified and its counterpart remains undetected, the possibility of a cryptic SV affecting the trans allele should always be considered. Apparent sequencing discrepancies, which are frequently dismissed as technical artefacts, may in fact represent subtle molecular signatures of hidden genomic rearrangements. Recognising and pursuing these signals can transform an inconclusive result into a definitive molecular diagnosis. This approach is particularly crucial in patients presenting with a well-defined clinical phenotype but incomplete molecular diagnoses, where reliance on SNV or CNV-based analyses alone may obscure pathogenic alleles of structural origin.

In summary, this study defines typical EV as a recessive hereditary cancer syndrome and demonstrates how the systematic integration of structural variant detection can resolve the hidden genetic architecture of rare, clinically well-defined disorders.

## Supplementary information

Supplementary material

Table S1

## Data Availability

Data supporting the study are available from the corresponding authors upon reasonable request.

## References

[1] Nagy R, Sweet K, Eng C. Highly penetrant hereditary cancer syndromes. Oncogene. 2004;23:6445–70. 15322516 10.1038/sj.onc.1207714

[2] Frebourg T, Bajalica Lagercrantz S, Oliveira C, Magenheim R, Evans DG. Guidelines for the Li-Fraumeni and heritable TP53-related cancer syndromes. Eur J Hum Genet. 2020;28:1379–86. 32457520 10.1038/s41431-020-0638-4 PMC7609280

[3] Georgiou D, Monje-Garcia L, Miles T, Monahan K, Ryan NA. A focused clinical review of Lynch syndrome. Cancer Manag Res. 2023;15:67–85. 10.2147/CMAR.S283668 PMC9868283 36699114

[4] Zaffaroni G, Mannucci A, Koskenvuo L, de Lacy B, Maffioli A, Bisseling T, et al. Updated European guidelines for clinical management of familial adenomatous polyposis (FAP), MUTYH-associated polyposis (MAP), gastric adenocarcinoma, proximal polyposis of the stomach (GAPPS) and other rare adenomatous polyposis syndromes: a joint EHTG-ESCP revision. Br J Surg. 2024;111:znae070. 38722804 10.1093/bjs/znae070 PMC11081080

[5] Leung AK, Barankin B, Lam JM, Leong KF, Hon KL. Xeroderma pigmentosum: an updated review. Drugs context. 2022;11:2022–2–5. 10.7573/dic.2022-2-5 PMC9045481 35520754

[6] Taylor AMR, Rothblum-Oviatt C, Ellis NA, Hickson ID, Meyer S, Crawford TO, et al. Chromosome instability syndromes. Nat Rev Dis Primers. 2019;5:64. 31537806 10.1038/s41572-019-0113-0 PMC10617425

[7] Weren RD, Ligtenberg MJ, Kets CM, De Voer RM, Verwiel ET, Spruijt L, et al. A germline homozygous mutation in the base-excision repair gene NTHL1 causes adenomatous polyposis and colorectal cancer. Nat Genet. 2015;47:668–71. 25938944 10.1038/ng.3287

[8] Al-Tassan N, Chmiel NH, Maynard J, Fleming N, Livingston AL, Williams GT, et al. Inherited variants of MYH associated with somatic G: C→ T: A mutations in colorectal tumors. Nat Genet. 2002;30:227–32. 11818965 10.1038/ng828

[9] Majewski S, Jabłońska S. Epidermodysplasia verruciformis as a model of human papillomavirus—induced genetic cancer of the skin. Arch Dermatol. 1995;131:1312–8. 7503577

[10] Lutzner MA, Blanchet-Bardon C, Orth G. Clinical observations, virologic studies, and treatment trials in patients with epidermodysplasia verruciformis, a disease induced by specific human papillomaviruses. J Investig Dermatol. 1984;83:S18–S25. 10.1111/1523-1747.ep12281128 6330217

[11] da Cruz Silva LL, de Oliveira WRP, Sotto MN. Epidermodysplasia verruciformis: revision of a model of carcinogenic disease. Surg Exp Pathol. 2019;2:20.

[12] Myers DJ, Kwan E, Fillman EP. Epidermodysplasia Verruciformis. [Updated 2024 Jul 20]. In: StatPearls [Internet]. Treasure Island (FL): StatPearls Publishing; 2024. 30480937

[13] Ramoz N, Rueda L-A, Bouadjar B, Montoya L-S, Orth G, Favre M. Mutations in two adjacent novel genes are associated with epidermodysplasia verruciformis. Nat Genet. 2002;32:579–81. 12426567 10.1038/ng1044

[14] De Jong SJ, Créquer A, Matos I, Hum D, Gunasekharan V, Lorenzo L, et al. The human CIB1–EVER1–EVER2 complex governs keratinocyte-intrinsic immunity to β-papillomaviruses. J Exp Med. 2018;215:2289–310. 30068544 10.1084/jem.20170308 PMC6122964

[15] Biglari S, Moghaddam AS, Tabatabaiefar MA, Sherkat R, Youssefian L, Saeidian AH, et al. Monogenic etiologies of persistent human papillomavirus infections: a comprehensive systematic review. Genet Med. 2024;26:101028. 37978863 10.1016/j.gim.2023.101028 PMC10922824

[16] Moore S, Rady P, Tyring S. Acquired epidermodysplasia verruciformis: clinical presentation and treatment update. Int J Dermatol. 2022;61:1325–35. 34403500 10.1111/ijd.15857

[17] Stenson PD, Mort M, Ball EV, Chapman M, Evans K, Azevedo L, et al. The Human Gene Mutation Database (HGMD®): optimizing its use in a clinical diagnostic or research setting. Hum Genet. 2020;139:1197–207. 32596782 10.1007/s00439-020-02199-3 PMC7497289

[18] Wu C-J, Li X, Sommers CL, Kurima K, Huh S, Bugos G, et al. Expression of a TMC6-TMC8-CIB1 heterotrimeric complex in lymphocytes is regulated by each of the components. J Biol Chem. 2020;295:16086–99. 32917726 10.1074/jbc.RA120.013045 PMC7681034

[19] Lazarczyk M, Dalard C, Hayder M, Dupre L, Pignolet B, Majewski S, et al. EVER proteins, key elements of the natural anti-human papillomavirus barrier, are regulated upon T-cell activation. PLoS ONE. 2012;7:e39995. 22761942 10.1371/journal.pone.0039995 PMC3386272

[20] Vuillier F, Gaud G, Guillemot D, Commere P-H, Pons C, Favre M. Loss of the HPV-infection resistance EVER2 protein impairs NF-κB signaling pathways in keratinocytes. PLoS ONE. 2014;9:e89479. 24586810 10.1371/journal.pone.0089479 PMC3929693

[21] Hasche D, Stephan S, Braspenning-Wesch I, Mikulec J, Niebler M, Gröne H-J, et al. The interplay of UV and cutaneous papillomavirus infection in skin cancer development. PLoS Pathog. 2017;13:e1006723. 29190285 10.1371/journal.ppat.1006723 PMC5708609

[22] Rehm TM, Parpoulas C, Straub E, Iftner T, Stubenrauch F. No evidence for restriction of Beta-HPV8 gene expression by epidermodysplasia verruciformis susceptibility genes CIB1, TMC6, or TMC8 in keratinocytes. Tumour Virus Res. 2025;20:200328. 10.1016/j.tvr.2025.200328 PMC12617823 41135646

[23] Wong M, Tu H-F, Tseng S-H, Mellinger-Pilgrim R, Best S, Tsai H-L, et al. MmuPV1 infection of Tmc6/Ever1 or Tmc8/Ever2 deficient FVB mice as a model of βHPV in typical epidermodysplasia verruciformis. PLoS Pathog. 2025;21:e1012837. 39813296 10.1371/journal.ppat.1012837 PMC11734914

[24] Torres AD, King RE, Uberoi A, Buehler D, Yoshida S, Ward-Shaw E, et al. Deficiency in Ever2 does not increase susceptibility of mice to pathogenesis by the mouse papillomavirus, MmuPV1. J Virol. 2024;98:e00174–00124. 38869286 10.1128/jvi.00174-24 PMC11265430

[25] Roche Sequencing Solutions. KAPA HyperCap Heredity Panel [Technical Data Sheet]. Basel (Switzerland): Roche; 2025.

[26] Van der Auwera GA, O’Connor BD. Genomics in the Cloud: Using Docker, GATK, and WDL in Terra. 1st ed. Sebastopol (CA): O’Reilly Media; 2020.

[27] Rausch T, Zichner T, Schlattl A, Stütz AM, Benes V, Korbel JO. DELLY: structural variant discovery by integrated paired-end and split-read analysis. Bioinformatics. 2012;28:i333–i339. 22962449 10.1093/bioinformatics/bts378 PMC3436805

[28] Dremsek P, Schwarz T, Weil B, Malashka A, Laccone F, Neesen J. Optical genome mapping in routine human genetic diagnostics—its advantages and limitations. Genes. 2021;12:1958. 34946907 10.3390/genes12121958 PMC8701374

[29] Richards S, Aziz N, Bale S, Bick D, Das S, Gastier-Foster J, et al. Standards and guidelines for the interpretation of sequence variants: a joint consensus recommendation of the American College of Medical Genetics and Genomics and the Association for Molecular Pathology. Genet Med. 2015;17:405–23. 25741868 10.1038/gim.2015.30 PMC4544753

[30] Riggs ER, Andersen EF, Cherry AM, Kantarci S, Kearney H, Patel A, et al. Technical standards for the interpretation and reporting of constitutional copy-number variants: a joint consensus recommendation of the American College of Medical Genetics and Genomics (ACMG) and the Clinical Genome Resource (ClinGen). 2020;22:245–257. 10.1038/s41436-019-0686-8 PMC7313390 31690835

[31] Karczewski KJ, Francioli LC, Tiao G, Cummings BB, Alföldi J, Wang Q, et al. The mutational constraint spectrum quantified from variation in 141,456 humans. Nature. 2020;581:434–43. 32461654 10.1038/s41586-020-2308-7 PMC7334197

[32] Landrum MJ, Lee JM, Benson M, Brown GR, Chao C, Chitipiralla S, et al. ClinVar: improving access to variant interpretations and supporting evidence. Nucleic Acids Res. 2018;46:D1062–D1067. 29165669 10.1093/nar/gkx1153 PMC5753237

[33] Kopanos C, Tsiolkas V, Kouris A, Chapple CE, Albarca Aguilera M, Meyer R, et al. VarSome: the human genomic variant search engine. Bioinformatics. 2019;35:1978–80. 30376034 10.1093/bioinformatics/bty897 PMC6546127

[34] Firth HV, Richards SM, Bevan AP, Clayton S, Corpas M, Rajan D, et al. DECIPHER: database of chromosomal imbalance and phenotype in humans using Ensembl resources. Am J Hum Genet. 2009;84:524–33. 19344873 10.1016/j.ajhg.2009.03.010 PMC2667985

[35] Desmet F-O, Hamroun D, Lalande M, Collod-Béroud G, Claustres M, Béroud C. Human splicing finder: an online bioinformatics tool to predict splicing signals. Nucleic Acids Res. 2009;37:e67–e67. 19339519 10.1093/nar/gkp215 PMC2685110

[36] McLaren W, Gil L, Hunt SE, Riat HS, Ritchie GR, Thormann A, et al. The Ensembl Variant Effect Predictor. Genome Biol. 2016;17:122. 27268795 10.1186/s13059-016-0974-4 PMC4893825

[37] Smit AFA, Hubley R, Green P. RepeatMasker Open-4.0. 2013–2015 http://www.repeatmasker.org.

[38] Imahorn E, Yüksel Z, Spoerri I, Gürel G, Imhof C, Saraçoğlu Z, et al. Novel TMC 8 splice site mutation in epidermodysplasia verruciformis and review of HPV infections in patients with the disease. J Eur Acad Dermatol Venereol. 2017;31:1722–6. 28646613 10.1111/jdv.14431

[39] Majewski S, Jabłońska S, Orth G. Epidermodysplasia verruciformis. Immunological and nonimmunological surveillance mechanisms: role in tumor progression. Clin Dermatol. 1997;15:321–34. 9255439 10.1016/s0738-081x(96)00169-1

[40] de Oliveira WR, Festa Neto C, Rady PL, Tyring SK. Clinical aspects of epidermodysplasia verruciformis. J Eur Acad Dermatol Venereol. 2003;17:394–8. 12834447 10.1046/j.1468-3083.2003.00703.x

[41] Gül U, Kiliç A, Gönül M, Cakmak SK, Bayis SS. Clinical aspects of epidermodysplasia verruciformis and review of the literature. Int J Dermatol. 2007;46:1069–72. 17910717 10.1111/j.1365-4632.2006.03014.x

[42] de Jong SJ, Imahorn E, Itin P, Uitto J, Orth G, Jouanguy E, et al. Epidermodysplasia verruciformis: inborn errors of immunity to human beta-papillomaviruses. Front Microbiol. 2018;9:1222. 29946305 10.3389/fmicb.2018.01222 PMC6005841

[43] Hastings PJ, Ira G, Lupski JR. A microhomology-mediated break-induced replication model for the origin of human copy number variation. PLoS Genet. 2009;5:e1000327. 19180184 10.1371/journal.pgen.1000327 PMC2621351

[44] Lee JA, Carvalho CM, Lupski JR. A DNA replication mechanism for generating nonrecurrent rearrangements associated with genomic disorders. Cell. 2007;131:1235–47. 18160035 10.1016/j.cell.2007.11.037

[45] Li Q, Mao Y, Li S, Du H, He W, He J, et al. Haplotyping by linked-read sequencing (HLRS) of the genetic disease carriers for preimplantation genetic testing without a proband or relatives. BMC Med Genom. 2020;13:117. 10.1186/s12920-020-00766-1 PMC7441613 32819358

[46] Godfred AC, Thomas Z, Peter D, Joseph A, Ravichandran L, George AA, et al. A novel large deletion in the EVER1 gene in a family with epidermodysplasia verruciformis from India. Am J Dermatopathol. 2024;46:373–6. 38574087 10.1097/DAD.0000000000002657

[47] Moore AR, Yu J, Pei Y, Cheng EWY, Taylor Tavares AL, Walker WT, et al. Use of genome sequencing to hunt for cryptic second-hit variants: analysis of 31 cases recruited to the 100,000 genomes project. J Med Genet. 2023;60:1235. 37558402 10.1136/jmg-2023-109362 PMC10715503

[48] Kvarnung M, Pettersson M, Chun-on P, Rafati M, McReynolds LJ, Norberg A, et al. Identification of biallelic POLA2 variants in two families with an autosomal recessive telomere biology disorder. Eur J Hum Genet. 2025;33:580–7. 39616267 10.1038/s41431-024-01722-8 PMC12048608

[49] Maia N, Soares G, Marques I, Rodrigues B, Santos R, Melo-Pires M, et al. Two compound heterozygous variants in SNX14 cause stereotypies and dystonia in autosomal recessive spinocerebellar ataxia 20. Front Genet. 2020;11:1038. 33193593 10.3389/fgene.2020.01038 PMC7543990

[50] Demidov G, Laurie S, Torella A, Piluso G, Scala M, Morleo M, et al. Structural variant calling and clinical interpretation in 6224 unsolved rare disease exomes. Eur J Hum Genet. 2024;32:998–1004. 38822122 10.1038/s41431-024-01637-4 PMC11291474

[51] Sanchis-Juan A, Stephens J, French CE, Gleadall N, Mégy K, Penkett C, et al. Complex structural variants in mendelian disorders: identification and breakpoint resolution using short- and long-read genome sequencing. Genome Med. 2018;10:95. 30526634 10.1186/s13073-018-0606-6 PMC6286558

[52] Collins RL, Brand H, Redin CE, Hanscom C, Antolik C, Stone MR, et al. Defining the diverse spectrum of inversions, complex structural variation, and chromothripsis in the morbid human genome. Genome Biol. 2017;18:36. 28260531 10.1186/s13059-017-1158-6 PMC5338099

[53] Stanganelli I, Spagnolo F, Argenziano G, Ascierto PA, Bassetto F, Bossi P, et al. The multidisciplinary management of cutaneous squamous cell carcinoma: a comprehensive review and clinical recommendations by a panel of experts. Cancers. 2022;14:377. 10.3390/cancers14020377 PMC8773547 35053539

