# Supplementary material for: TMC6/8-associated epidermodysplasia verruciformis: germline variants and a complex structural alteration in a skin cancer predisposition syndrome
Source: Eur J Hum Genet. 2026 Feb 18;34(3):429–37. doi: 10.1038/s41431-026-02043-8 (PMC12963407; doi:10.1038/s41431-026-02043-8)
Supplement: Supplementary file 1 — Supplementary material [file 41431_2026_2043_MOESM1_ESM.docx]

**The proposed replication-based mechanism underlying this complex rearrangement:**

OGM elucidated the structural variant hypothesized based on prior sequencing results, resolving it as a complex deletion–inversion spanning both *TMC6* and *TMC8* in cis on a single chromosome. Because the structural variant also affected the *TMC6* locus, the possibility of an additional point mutation in *TMC6* was re-evaluated but excluded. Long-range PCR across the OGM-predicted breakpoints, followed by Sanger sequencing, further refined the configuration of the complex SV as a del–inv–del variant that also included a 16-bp deletion within TMC8 intron 14. Breakpoint-level sequencing revealed short sequence overlaps: a C-tract microhomology (CCCC/CCCCC) at the first junction, associated with deletion of the intervening TMC8 segment, and a minimal TG similarity at the second junction. The junction structure observed in P6 suggests a replication-based mechanism such as fork stalling and template switching (FoSTeS) or microhomology-mediated break-induced replication (MMBIR). Breakpoint junctions in replication-based rearrangements typically display very short sequence overlaps, often only 2–5 bp, which are sufficient to stabilize transient template switching ^1^. The short sequence overlaps identified at the TMC6/TMC8 breakpoints in P6 fall within this range and provide a plausible mechanistic basis for the observed del–inv–del configuration. We propose that replication fork stalling occurred within the C-tract of TMC8 intron 14, a sequence prone to secondary structure formation and polymerase pausing. Following fork collapse, the lagging strand disengaged from the stalled TMC8 template and annealed to a nearby region in TMC6 intron 18 through short C-tract microhomology, forming the first breakpoint. DNA synthesis then resumed on the TMC6 template, deleting the intervening 16-bp TMC8 segment. A subsequent template switch from TMC6 intron 13 back to TMC8 intron 14 restored the original orientation and completed the rearrangement. The sequential occurrence of these two template switches produced the complex TMC6/TMC8 del–inv–del configuration, encompassing a multi-exonic TMC6 deletion and a 16-bp TMC8 microdeletion. This model provides a coherent mechanistic explanation for the complex structural variant and exemplifies how replication-based repair processes can generate composite rearrangements from short or even negligible sequence overlap.

1. Nazaryan-Petersen, L. *et al.* Replicative and non-replicative mechanisms in the formation of clustered CNVs are indicated by whole genome characterization. *PLoS Genet* **14**, e1007780 (2018).
