# Supplementary material for: TMC6/8-associated epidermodysplasia verruciformis: germline variants and a complex structural alteration in a skin cancer predisposition syndrome
Source: Eur J Hum Genet. 2026 Feb 18;34(3):429–37. doi: 10.1038/s41431-026-02043-8 (PMC12963407; doi:10.1038/s41431-026-02043-8)
Supplement: Supplementary file 2 — Table S1 [file 41431_2026_2043_MOESM2_ESM.docx]

| **Sample ID** | **Gene** | **% Bases ≥ 5x** | **% Bases ≥ 20x** |
| --- | --- | --- | --- |
| **Patient 6 (Proband)** | *TMC6* | 100% | 94% |
|  | *TMC8* | 100% | 92.3% |
|  | *CIB1* | 100% | 100% |

**Table S1. Sequencing coverage metrics for *TMC6*, *TMC8*, and *CIB1* genes in Proband P6.**

Table S1. Sequencing coverage metrics for TMC6, TMC8, and CIB1 in proband P6.

Coverage is shown as the percentage of targeted bases achieving ≥5× and ≥20× read depth in CES.

Note: Other affected individuals in the cohort were evaluated by targeted Sanger sequencing.
